# Enhanced formulation and comprehensive analysis of novel natural ointments with grape seed and pomegranate peel infused in olive oil

**DOI:** 10.55730/1300-0152.2721

**Published:** 2024-12-16

**Authors:** Mebarka Imane BENGUECHOUA, Madjda BENGUECHOUA, Khedidja BENAROUS, Alaeddine KAOUKA, Mohamed YOUSFI

**Affiliations:** 1 Applied Sciences and Didactics Laboratory, Higher Normal School, Laghouat, Algeria; 2 Fundamental Sciences Laboratory, Amar Telidji University, Laghouat, Algeria

**Keywords:** Grape and pomegranate byproducts, infused olive oil, cosmetic formulations, antioxidant, skincare

## Abstract

**Background/aim:**

The global shift toward sustainable and organic alternatives in cosmetics and personal care has created a demand for innovative and environmentally conscious formulations. This study aimed to develop novel natural ointments using beeswax and grape seed (GS) and pomegranate peel (PP) extracts infused in extra virgin olive oil (EVOO), and determine the physicochemical characteristics, microbiological properties, and antioxidant activity as a way to valorize organic waste toward its application in the cosmetic industry. The research aimed to promote sustainability and effectiveness in the cosmetic industry.

**Materials and methods:**

Microbial analysis was performed on the oils and ointments, followed by the extraction of active compounds using dimethyl sulfoxide (DMSO). The chemical profiles of the extracts were analyzed through thin-layer chromatography, while gas chromatography-mass spectrometry (GC-MS) was employed for compound identification and quantification. In vitro assays were conducted to assess the antioxidant activity of the extracts. The physical properties of the formulated ointments (organoleptic properties, homogeneity, pH, water resistance, skin absorption, topical sensitivity, and stability) were evaluated for potential topical applications.

**Results:**

The DMSO extracts from the GSs and PP-infused EVOO exhibited significant antioxidant activity. The GC-MS analysis revealed an increase in oleic acid content, ranging from 61.46% in pure EVOO to 72.49% in the infused samples, enhancing their suitability for skin health. The ointments demonstrated favorable physical properties. Microbial analysis confirmed no detectable contamination, ensuring the safety of the formulations for topical use.

**Conclusion:**

The EVOO-based ointments enriched with GS and PP extracts exhibited strong antioxidant activity, stability, water resistance, and moisturizing properties. The use of phenolic compounds and natural ingredients like beeswax and olive oil underscores the potential of agricultural byproducts in creating sustainable, ecofriendly cosmetics, with promising applications in skincare and therapy.

## 1. Introduction

Olive oil, renowned for its rich array of vitamins and antioxidants, offers remarkable benefits for skin care, especially when combined with beeswax. This powerful duo not only deeply moisturizes, but also creates a protective barrier that locks in hydration while allowing the skin to breathe (González-Acedo et al., 2023). Olive oil’s antiinflammatory properties help soothe irritation and promote healing, making it ideal for sensitive or sun-damaged skin (Alnemer et al., 2021).

Olive oil contains an abundant amount of oleic acid, a monounsaturated fatty acid (MUFA) known for its moisturizing and antiinflammatory properties, making it an excellent skincare ingredient (González-Acedo et al., 2023). Its phenolic compounds, including hydroxytyrosol and oleuropein, offer antioxidant benefits that help combat skin aging (Alnemer et al., 2021). Additionally, olive oil is rich in vitamins E and A, which promote skin regeneration and healing (Alnemer et al., 2021; González-Acedo et al., 2023).

In spa treatments, olive oil and beeswax are often used in luxurious body wraps and massages, enhancing relaxation while nourishing the skin (González-Acedo et al., 2023; Juncan et al., 2023). Together, they provide a natural solution for achieving a radiant, youthful complexion, making them staples in holistic skin care regimens (Juncan et al., 2023)

Beeswax, a natural secretion from worker bees, has found diverse applications in modern society. In skincare, its versatility shines as an occlusive agent, helping to maintain skin hydration by minimizing water loss (Nong et al., 2023). Additionally, beeswax acts as a humectant, drawing moisture into the skin, and an emollient, providing a soothing and softening effect (Nong et al., 2023). As a natural substance, beeswax has been shown to alleviate symptoms associated with common skin conditions, such as dermatitis, psoriasis, and imbalances in skin flora (Nong et al., 2023).

Fruits and vegetables are the horticulture products that are consumed the most and comprise waste items like peels and seeds. Food waste valorization is the process of utilizing food waste as a resource to create new products, rather than discarding it. Food waste can provide a valuable feedstock for the recovery of bioactive compounds, including antioxidants and dietary fiber. These bioactive compounds have been shown to have health benefits, including antioxidant, antibacterial, antiinflammatory properties, and skin care (Girotto et al., 2015; Calcio Gaudino et al., 2020; Grillo et al., 2020; Cravotto et al., 2022).

Ancient civilizations, including the Egyptians and Greeks, utilized grape seeds (GSs) in poultices for treating skin conditions due to their antiinflammatory properties (Zhu et al., 2014). Similarly, pomegranate peels (PPs) have been valued since antiquity for their medicinal benefits, including antioxidant and antimicrobial effects, which are now supported by modern research (George et al., 2019; Castro et al., 2023).

Grapes, the berries of *Vitis vinifera L* of the family Vitaceae, are among the most widely cultivated fruit crop globally, with approximately 75 million tons produced annually (George et al., 2019). Grape pomace, a byproduct of grape processing, is primarily composed of seeds, skins, and stems (Zhu et al., 2014). GS extract has emerged as a powerhouse ingredient in skin care, celebrated for its numerous benefits backed by recent research. Rich in polyphenols, particularly proanthocyanidins, it exhibits potent antiaging properties by reducing wrinkles and enhancing skin elasticity (Sochorova et al., 2020). Its strong antioxidant activity protects skin from oxidative stress caused by ultraviolet (UV) radiation and environmental pollutants, while also promoting wound healing through increased vascular endothelial growth factor production (Castro et al., 2023). Additionally, GS oil serves as an effective emollient, locking in moisture without a greasy feel, and its antiinflammatory effects help soothe irritated skin conditions such as acne and eczema. These multifaceted benefits make GS extract a valuable addition to modern skin care formulations, contributing to healthier, more resilient skin (Sochorova et al., 2020 ; Castro et al., 2023).

GSs are rich in linoleic acid, an essential fatty acid that enhances emollient properties and supports the skin barrier. They also contain polyphenols, particularly proanthocyanidins, which provide strong antioxidant protection, reducing oxidative stress and UV damage. Additionally, condensed tannins in GSs contribute antimicrobial and antiinflammatory benefits (Kennedy et al., 2000; Sabir et al., 2012; Sorolla et al., 2018).

Pomegranate, scientifically known as *Punica granatum* L., is a fruit-bearing tree that belongs to the family Lythraceae. Indigenous to the Mediterranean region, pomegranate has a long history of use in traditional medicine, with its natural antiviral, antifungal, and antibacterial properties making it a valuable resource for treating a variety of ailments (Das et al., 1999). Pomegranate fruit is a significant source of phenolic substances, with the peel alone accounting for approximately 50% of the fruit’s weight and containing a wide array of bioactive compounds (George et al., 2019). These compounds include flavonoids, tannins, and phenolics with antimicrobial properties, which have been shown to exhibit potent antioxidant activities (George et al., 2019). Pomegranate peels (PPs) contain a variety of bioactive substances, including quercetin, catechin, antioxidants, minerals, and vitamins, as well as several potential health benefits (Pirzadeh et al., 2021). PPs have gained recognition in skin care for their impressive array of benefits, supported by recent research. These peels effectively combat oxidative stress, which is crucial for preventing premature aging and reducing the appearance of wrinkles and fine lines (Mo et al., 2022; Ain et al., 2023). Studies have shown that the ellagic acid found in PPs stimulates collagen production, enhancing skin firmness and elasticity. Additionally, their antimicrobial and antiinflammatory properties make them effective in treating acne and soothing irritated skin conditions. PP extracts have also demonstrated potential in improving hyperpigmentation and promoting wound healing, making them a valuable ingredient in cosmetic formulations aimed at achieving a brighter, healthier complexion. Overall, the incorporation of PPs into skin care routines offers a natural solution for enhancing skin health and appearance (Mo et al., 2022; Ain et al., 2023).

An ointment is a semisolid topical formulation primarily composed of oil, designed for application to the skin. It typically has a greasy consistency and serves to protect or heal the skin by delivering therapeutic substances directly to affected areas (Kaushal and Upadhyaya, 2022). The use of organic ointments formulated with herbal drugs has grown rapidly in recent years. This trend can be explained by several factors such as organic ointments are made from natural ingredients known for their beneficial properties for the skin, as well as the support for the local economy (Wachtel-Galor and Benzie, 2012; Ekor, 2014).

The current research bridges the gap between waste management and skincare innovation, paving the way for ecoconscious formulations that meet consumer demands for natural and effective skincare products. This study proposed the development of a novel, organic ointment formulated with GS and PP extract infused in olive oil, and beeswax, which was analyzing to determine the physicochemical characteristics. The objective was to create a sustainable, topical product that offers deep hydration, and protects the skin from environmental stressors.

## 2. Material and methods

### 2.1. Chemicals and reagents

1,1-Diphenyl-2-picrylhydrazyl radical (DPPH, purity ≥99%), quercetin (≥99%), catechin (≥98%), and rutin (≥95%) were purchased from Sigma-Aldrich (St. Louis, MO, USA). All the other chemicals and reagents, including methanol, ethyl acetate, and sodium carbonate, were of analytical grade and sourced from certified suppliers.

### 2.2. Materials

The grapes were purchased directly from a market in Laghouat, Algeria, where grape production plays a significant role in the local agricultural sector, and the pomegranates were obtained from a farm in Laghouat Province, south of Algeria. The GSs and PPs were air-dried in shaded areas at room temperature for a week before being coarsely ground into powder using a dry grinder. The resulting powder was then stored in airtight bags, in the dark, for future use. In the maceration process, extra virgin olive oil (EVOO) was obtained from Dar Diaf, a certified organic olive oil producer in Algeria. Beeswax, sourced from a local beekeeper, was used as one of the natural raw materials in the ointments.

### 2.3. Preparation of the GS and PP-infused EVOO

**Figure 1:**
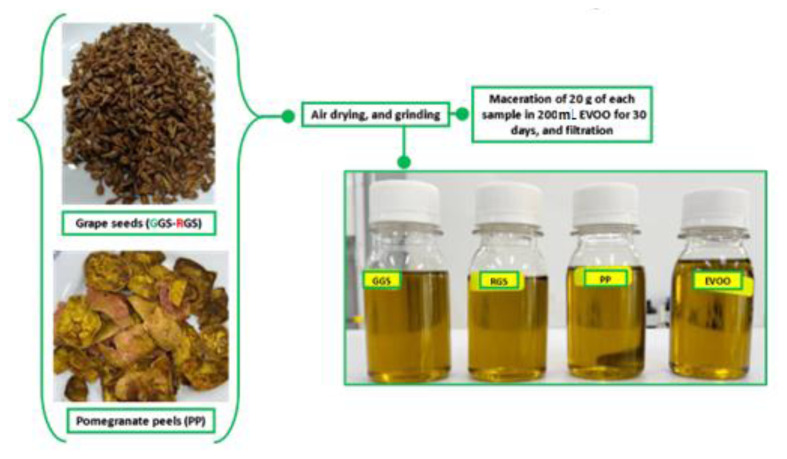
Process used for infusing the GSs and PPs with the EVOO.

The protocol for preparing the GS and PP-infused EVOO is shown in Figure 1, as mentioned in our previous work with slight modifications (Benguechoua et al., 2024).

The maceration process was carried out for 30 days, in the dark, at room temperature (Eroglu and Girgin, 2021; Benguechoua et al., 2024)

The GS and PP-infused EVOO was meticulously preserved in bottles, in the dark, at room temperature until they were ready for use.

#### 2.3.1. Physical analysis of the oil samples (refractive index)

The refractive index of the infused samples was determined using a refractometer following the AOAC (2000) (The Association of Official Analytical Chemists, Gaithersburg, MD, USA) (Osei, 2013).

### 2.4. Phytochemical analyses

#### 2.4.1. Extraction with DMSO

The methods by Eroglu and Girgin (2021) and our previous work (Benguechoua et al., 2024) were modified to prepare the extract. First, a precise volume of dimethyl sulfoxide (DMSO) was measured and combined with double the volume of GS and PP-infused EVOO in a beaker. The mixture was then gently stirred on a hot plate at 37 °C for 2 h. After resting undisturbed in the dark for 1 h, the mixture separated into two distinct layers. A clear lower layer of DMSO had formed, while the GS and PP-infused EVOO had settled as an upper layer. The DMSO layer was carefully transferred into a glass tube for further processing. For comparison, a control test tube containing only EVOO underwent the same extraction process. Finally, all the extracts dissolved in DMSO were stored promptly in a refrigerator for future use. Common characterization methods, including colored tube tests, were used to identify the main groups of secondary metabolites present and thin-layer chromatography (TLC) analysis.

#### 2.4.2. Phenolic compound analysis

To detect the phenolic content of all the DMSO extracts, the Folin-Ciocalteu test was employed, as established by previous studies (Maiga et al., 2020). Following the standard protocol, 100 μL of each extract sample was combined with 250 μL of Folin-Ciocalteu reagent in a test tube. This mixture was incubated for 2 min at room temperature. Next, 1000 μL of sodium carbonate (Na_2_CO_3_) solution was added and the samples were incubated again for 30 min in the dark at room temperature. The development of a blue color indicated the presence of phenolic compounds in the extracts.

#### 2.4.3. Flavonoid analysis

The AlCl_3_ test (Dirar et al., 2019) was employed to assess the flavonoid content of the DMSO extracts. In this test, 500 μL of each extract sample was combined with 500 μL of a 10% AlCl_3_ solution in separate test tubes. The mixtures were then incubated at room temperature for 15 min. The development of a yellow color served as an indication of the presence of flavonoids in the extracts.

#### 2.4.4. TLC analysis

TLC was performed using Merck (Merck KGaA, Darmstadt, Germany) high-performance TLC plates (20 × 10 cm) coated with silica gel 60 F254. The mobile phase for the separation consisted of a mixture of ethyl acetate, methanol, and water at a ratio of 10:1.35:1 (v/v/v) (Benarous et al., 2015). Standards of quercetin, catechin, and rutin (each at a concentration of 2.5 mg/mL) were applied alongside the DMSO extracts as separate strips on the TLC plates. After the development process (where the mobile phase separates the components), the plates were dried using a cold air stream from a dryer. Finally, the plates were observed under UV light at both 254 and 366 nm using a camera. After spotting the samples, the TLC plate was then dipped in a TLC chamber with crystals of iodine (I_2_) to reveal the separated components. This developer causes the molecules to appear as brown spots on the plate. The ability of the sample components to neutralize free radicals was then visualized using DPPH, a stable free radical molecule (Wagner and Bladt, 1996; Maleš et al., 2004). After spraying the plate with a DPPH solution and allowing it to dry, zones with antioxidant activity appeared as yellow spots against the dark purple background. These zones were photographed under visible light using a professional camera. Additionally, for each separated component, retention factor (R_f_) values were calculated based on methods described in various scientific works (Wagner and Bladt, 1996; Maleš et al., 2004).

### 2.5. Fatty acid composition

The fatty acid methyl esters (FAMEs) were prepared according to the following procedure (Benguechoua et al., 2021): Initially, 0.5 g of the different oils (EVOO, green GS (GGS), red GS (RGS), and PP) were refluxed with 20 mL of 0.5% sodium methylate (NaOMe) for 30 min. Once cooled, 20 mL of distilled water was added to the mixture. The FAMEs were then extracted using dichloromethane, washed with distilled water, and dried over anhydrous Na_2_SO_4_. Finally, after filtration and evaporation of the dichloromethane, the FAMEs were purified. The analysis of the FAMEs was performed using a Shimadzu gas chromatography-mass spectrometry (GCMS)-QP2020 instrument (Shimadzu Corp., Kyoto, Japan) fitted with a Rxi-5ms capillary column (30 m × 0.25 mm, film thickness of 0.25 μm). A sample volume of 0.3 μL, diluted in n-hexane, was injected in split mode (1:80) at 250 °C, while the detector was maintained at 330 °C. The temperature program for the column began at 70 °C for 1 min, increased to 160 °C at a rate of 15 °C/min, held for an additional 2 min, then ramped up to 260 °C at 7 °C/min and held for 5 min, before finally being increased to 330 °C at a rate of 5 °C/min and held for 10 min. Helium (99.995% purity) served as the carrier gas with a flow rate of 1 mL/min. The mass spectrometer operated under conditions of an ionization voltage of 70 eV and an ion source temperature of 200 °C, acquiring electron ionization mass spectra across a range of 45–600 m/z.

### 2.6. Ointment preparation

Ointments were formulated by incorporating GS and PP-infused EVOO into beeswax (2 g). The beeswax was melted at 65 °C, followed by the addition of the oils. The solution was gently heated until homogenous, then allowed to cool to form an ointment with optimal consistency. The prepared ointments were stored for subsequent analysis. This simple but efficient method successfully incorporated the GS and PP-infused EVOO into a stable ointment with a pleasant texture. The careful heating and cooling steps guaranteed a uniform mixture and the desired consistency in the final ointments.

#### 2.6.1. Analysis of the physical properties of the ointments

Several physical properties were analyzed, such as the organoleptic properties, homogeneity, water resistance, skin absorption, and skin sensitivity, to ensure the quality, stability, and user experiences with the GS- and PP-based ointments.

#### 2.6.2. Organoleptic properties

The appearance, color, odor, and texture of the ointments were also evaluated to confirm that they meet sensory requirements.

#### 2.6.3. Homogeneity analysis

The consistency of active ingredient distribution within the ointments was evaluated by assessing their homogeneity. A 0.5-g sample of the ointment was placed on a smooth surface and spread into a thin layer. The ointment was visually inspected for uniform color and the absence of lumps or granules. This inspection ensured that the active ingredients were evenly dispersed throughout the ointments, meeting the standards for consistent therapeutic efficacy.

#### 2.6.4. pH analysis

The pH of the ointments was assessed to ensure that they fell within the suitable range for topical use, ideally aligning with the skin’s natural pH. A thin layer of the ointment was applied to pH paper, and it was allowed to stand for a brief period. The pH level was subsequently determined by comparing the color change to a standardized chart.

#### 2.6.5. Water resistance analysis

The ointments were evaluated in terms of their water resistance, a crucial property for topical products to maintain their integrity and efficacy when exposed to moisture. A thin layer of each ointment (1 g) was applied to a glass plate, and a drop of water was then added. If the ointment and water remained immiscible, it indicated that the formulation possessed the desired water-resistant characteristics, ensuring the product’s stability and performance when applied to the skin. Three trials were performed to ensure the reproducibility and accuracy of the results.

#### 2.6.6. Skin absorption analysis

The ointments underwent assessment for their skin absorption capability, which is essential for effectively delivering active ingredients to the intended site. First, 1 g of the ointment was applied to a 4-cm circular area on the hand, followed by circular motions to facilitate absorption. The duration required for the ointment to be absorbed was carefully noted. This process was repeated three times for each type of ointment to ensure consistency and reliability in the evaluation of skin absorption properties (Zsikó et al., 2019; Adamiak-Giera et al., 2023).

#### 2.6.7. Topical sensitivity analysis

The ointments were thoroughly analyzed for any potential skin irritation or sensitization reactions to ensure that they were well-tolerated when applied topically. All the ointments underwent comprehensive skin sensitivity assessment. First, 1 g of each ointment was applied to the skin for a week, and the test subjects were closely monitored for any adverse reactions, such as skin inflammation, irritation, reddening, or other allergic responses. This rigorous evaluation process allowed for the assessment of the safety and suitability of the ointments for topical application, prioritizing the comfort and well-being of the users (Naeimifar et al., 2020; Ganguly et al., 2022).

#### 2.6.8. Microbial assessment and preservation of the ointments

The antimicrobial protection properties of the ointments was assessed using established protocols, including the enumeration and detection of aerobic mesophilic bacteria (SR EN ISO 21149: 2017), yeast and mold counts (SR EN ISO 16212: 2017), and detection of *Staphylococcus aureus* (SR EN ISO 22718: 2016), *Candida albicans* (SR EN ISO 18416: 2016), *Escherichia coli* (SR EN ISO 21150: 2016), and *Pseudomonas aeruginosa* (SR EN ISO 22717: 2016) (Juncan et al., 2023).

#### 2.6.9. Stability analysis of the ointments

The accelerated stability study was performed for a period of 6 months under conditions of product storage at 4 °C and at ambient temperature.

## 3. Results and discussion

### 3.1. DMSO extraction of the GS and PP-infused EVOO

The analysis of phytochemicals in plant-based products, particularly in oil seed plant extracts with complex compositions, continues to be a significant challenge. Previous research commonly used methanol as the preferred solvent to extract bioactive compounds from plant-infused oils in order to perform phytochemical characterization (Orhan et al, 2013; Heinrich et al., 2017). This study demonstrated the effectiveness of DMSO in extracting bioactive compounds from EVOO infused with GS and PP. The method successfully yielded extracts containing bioactive components from the infused EVOO. Interestingly, the transparent DMSO solution changed color based on the specific infused olive oil, offering a potential visual indicator during the extraction process. Optimizing extraction conditions, including the incubation time, darkness, and temperature, plays a crucial role in minimizing oxidation. This, in turn, helps preserve the organoleptic properties (sensory characteristics) of the oil, potentially leading to superior quality compared to studies using less controlled methods (Eroglu and Girgin, 2021).

### 3.2. Qualitative phytochemical screening

Preliminary analysis of the DMSO extracts using phytochemical screening revealed a wealth of bioactive components, including phenolics and flavonoids. These extracts demonstrated a strong presence of phenolics and flavonoids, as evidenced by positive test results (+++++) for all the samples, which could be due to the enhanced solubility of these active compounds in organic solvents (Figure S1).

### 3.3. TLC analysis

**Figure 2:**
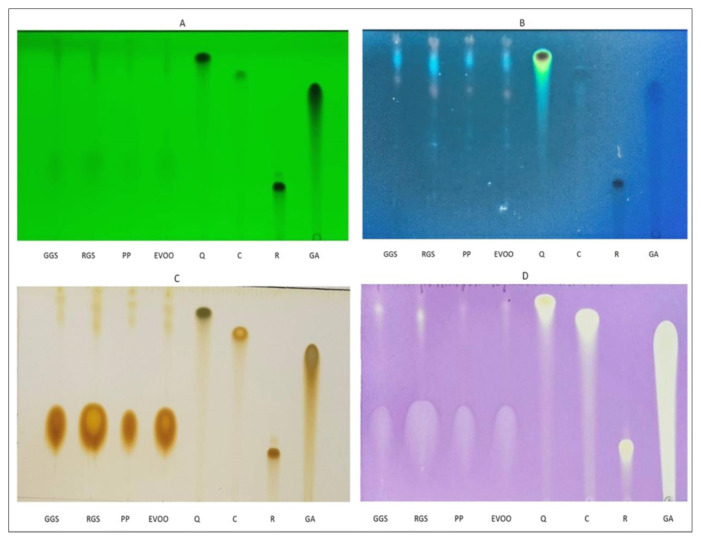
TLC chromatograms of DMSO-GSs and PP-EVOO, DMSO-EVOO extracts, and standards at 254 nm (A), 366 nm (B), in iodine (C), and under white light after DPPH assay (D).

For the accurate analysis of antioxidant compounds using TLC-DPPH, a two-step process is recommended after spraying with DPPH solution: drying the TLC plate followed by immediate exposure to white light. This efficient approach ensures the formation of stable colored end products, minimizing the risk of misinterpretations. The UV detection of antioxidant spots further strengthens their identification. These spots only exhibit fluorescence at 366 nm (Figure 2A), remaining invisible at 254 nm (Figure 2B). This contrasting behavior suggests the presence of unique chemical functionalities associated with antioxidant activity. Treatment with the iodine developer solution yielded brown spots on the TLC plate, corresponding to the locations of the separated molecules (Figure 2C). Based on these spots R_f_ values, which matched those of quercetin (0.86), catechin (0.78), rutin (0.24), and gallic acid (0.70), the presence of these compounds was determined on all the TLC plates (Table 1). Analysis using standard compounds revealed the presence of quercetin and catechin in all the DMSO extracts obtained from the GS and PP**-**infused EVOO. UV visualization of the TLC plates at 366 nm over time revealed the presence of multiple fluorescent spots in all the extracts. These spots exhibited varying intensities, suggesting differences in polarity and potentially unique chemical properties. The iodine-developed TLC plate (Figure 2C) offers a visual map of the phenolic compounds present in the plant extracts. By separating these compounds based on their polarity, the TLC plate revealed the presence of a diverse range of phenolics with varying characteristics.

Analysis of the iodine-developed TLC plates revealed the presence of four distinct spots in all the DMSO extracts containing GSs and PPs and EVOO, the R_f_ values for these spots ranged from 0.26 to 0.96 (Table 1). The success of the extraction process was confirmed by the presence of positive antioxidant activity in the extracts. This was determined by monitoring the transformation of the purple DPPH solution to a yellow color, a well-established method for assessing antioxidant capacity. Further analysis using TLC-DPPH revealed the presence of several distinct zones within the DMSO-GS and PP-EVOO, and DMSO-EVOO extracts, potentially corresponding to various antioxidant compounds.

GS extracts with a wide range of condensed tannin contents, from 15% up to 90%, are commercially available for use in oenology, cosmetics, and phytotherapy. However, these products often lack detailed information about the characteristics of the raw material used. In general, the most interesting and abundant compounds found in GSs are polyphenols, particularly condensed tannins, as well as fatty acids (Kennedy et al., 2000; Sorolla et al., 2018).

A study by Toledo-Merma et al. (2022) on pomegranate showed the presence of ellagic acid with an R_f_ = 0.83 (Toledo-Merma et al., 2022). Other studies conducted both in vitro and in vivo have highlighted the diverse range of biological and health benefits associated with compounds found in PPs. These benefits include antioxidant, antiinflammatory, antimutagenic, anticarcinogenic, and antihypertensive properties. Moreover, these compounds have shown promise in addressing various health conditions such as cardiovascular problems, diabetes, and obesity (Kadam et al., 2018; Kandylis and Kokkinomagoulos, 2020).

### 3.4. Physical analysis of the GS and PP-infused EVOO samples

Table 1 summarizes the refractive index of the GS and PP-infused EVOO samples. The refractive index of the infused EVOO samples remained within the acceptable range (1.4659–1.4665) established by Codex Alimentarius (2017) for olive oil. This suggests minimal impact on the core quality parameters associated with olive oil.

### 3.5. Fatty acid composition

The results of GC-MS analysis of the FAMEs of the EVOO, and GGSs, RGSs, and PP-infused EVOO are shown in Table 2. In all the samples, the main saturated fatty acids detected were palmitic acid (C16:0 = 12.95%–14.62%) followed by stearic acid (C18:0 = 2.67%–3.51%). On the other hand, the main unsaturated fatty acids were oleic acid (C18:1 = 61.46%–72.49%), followed by linoleic acid (C 18:2 = 7.52%–8.7%) and palmitoleic acid (C16:1 = 0.79%–1.09%), although others were found to be of low overall abundance.

The fatty acid composition of the GGS, RGS, PP, and EVOO revealed the predominance of two primary fatty acids, oleic acid (C18:1) and palmitic acid (C16:0), which collectively represented over 80% of the total fatty acid profile in the infused oils (GGS, RGS, and PP) (Table 2). Furthermore, analyses indicate the presence of minor quantities of additional fatty acids, such as palmitoleic acid (C16:1), linoleic acid (C18:2), and stearic acid (C18:0). These findings underscore the significant contributions of these fatty acids to the overall composition of the examined oils. According to the results in Table 2, the GS and PP significantly modified the chemical composition of olive oil, notably increasing the oleic acid content, which offers multiple benefits for skin health due to its moisturizing, antiinflammatory, and antioxidant properties, making it an ideal ingredient to treat various skin problems, including dryness, irritation, and signs of aging. These results are consistent with the study conducted by Kapcsándi et al. (2021) on GSs. Regarding the two GS varieties, significant differences were found in the fatty acid composition of the oils of each grape variety, a result supported by several studies (Sabir et al., 2012; Konuskan et al., 2019). PP provides powerful antioxidant and antiinflammatory effects when infused into olive oil. These properties help protect the skin from oxidative stress and inflammation, making the infused oil beneficial for various skin conditions such as acne, eczema, and psoriasis (Zielińska et al., 2022; Singh et al., 2023).

The area of some peaks identified for fatty acids is reduced or disappears when GSs or PPs are added to olive oil. This suggests that GSs or PPs may interact physically and chemically with some of the fatty acids present. These interactions may be influenced by the porous structure and high specific surface area of GSs and PPs, which provide numerous adsorption sites for fatty acid molecules, or by the surface properties of GSs and PPs, such as polarity and charge, which favor the selective adhesion of certain types of fatty acids, or by operating conditions such as temperature, pressure, and contact time, which modulate the efficiency of these interactions (Abd EL-khalik and Omar, 2018; De Souza et al., 2020). Olive oil is rich in oleic acid, a MUFA that has emollient and softening properties for the skin, and it continues to nourish the epidermis in depth. Its moisturizing properties help to keep the skin supple and prevent skin dryness, although the addition of GSs and PPs slightly modifies the composition of olive oil, with an improvement in its main beneficial properties for the skin: hydration, nutrition, regeneration, and antioxidant protection (Alnemer et al., 2022).

### 3.6. Analysis of the physical properties of the ointments

#### 3.6.1. Organoleptic analysis

**Figure 3:**
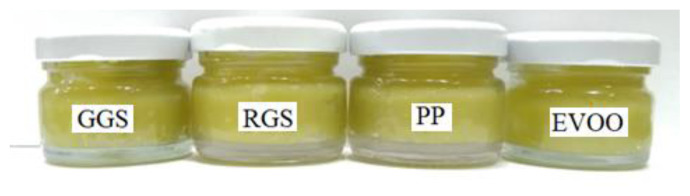
Formulated ointments.

Organoleptic characteristics refer to sight, smell, taste, and touch. These sensory aspects combine to create a product’s organoleptic characteristics. For the GS and PP-infused EVOO ointments, the type of GSs and PPs and the quality of the olive oil used in an ointment can significantly impact how the user perceives the final product. The synergistic effects of the EVOO, GSs, and PPs in the ointments yielded a distinctive product with a rich, light green color and a moderately heavy, emollient texture. This combination also imparted a pleasant aroma, making it a desirable choice for addressing various skin concerns (dry skin, itchiness, inflammation, etc.). The taste and aroma of the ointment were primarily influenced by the olive oil flavor, which is a characteristic of EVOO. This distinct flavor profile is the result of the careful selection and blending of high-quality olive oil, ensuring a consistent and pleasant sensory experience for users. The ointments are represented in Figure 3.

#### 3.6.2. Homogeneity analysis

A critical quality analysis for any ointment is homogeneity, which guarantees a consistent product throughout. To assess this, the ointments were visually inspected for uniform color and the absence of lumps or granules. The visual homogeneity analysis confirmed that all the ointments made with GS and PP-infused EVOO maintained a uniform appearance both before and after application.

#### 3.6.3. pH analysis

Healthy skin has a delicate balance, like a shield protecting from germs. This shield is slightly acidic, typically around a pH of 5 to 6 (Souto et al., 2022). To keep skin healthy, ointments should also be close to this pH. That is why checking the pH of ointments is so important. It ensures that they will not disrupt the skin’s natural defense system and keeps them working effectively and safely. The skin’s pH level is a delicate balance that plays a crucial role in its overall health and function. Disruptions to this pH, especially toward a more alkaline state, can have significant consequences for the skin’s barrier and overall wellbeing. Ointment formulations must be carefully designed to support and preserve the skin’s natural pH equilibrium. The pH values of all the formulated ointments were between 5 and 6. This matches the natural acidity of skin, which helps minimize irritation when applied.

#### 3.6.4. Water resistance analysis

All the ointments were devoid of water and consisted of components that were insoluble in water. This composition resulted in a nonmiscible and nonabsorbent interaction with water. Anhydrous ointments exhibit superior water resistance compared to water-based emulsions, allowing them to remain on the skin surface for extended periods without drying out.

Upon topical application, these ointments created a protective barrier on the surface of the skin. This barrier effectively limited the natural water evaporation from the skin, thereby enhancing its hydration levels. By forming this barrier, anhydrous ointments help maintain the skin’s moisture, promoting skin health and overall hydration.

#### 3.6.5. Skin absorption

For topical medications to be effective, they need to reach the deeper layers of the skin. Ointments excel at this because they are oil-based, they can penetrate and deliver the medicine right where it is needed. Several variables influence the absorption of ointments into the skin, encompassing aspects such as the cornified layer, particle size of the medication, skin hydration levels, duration of contact, skin temperature, and any existing epidermal damage. Of these factors, the cornified layer, which constitutes the outermost stratum of the epidermis, plays a pivotal role in governing the absorption of ointments (Lambers et al., 2006). Increasing skin moisture levels can boost the absorption of medication by softening the cornified layer, facilitating more effective penetration of the ointment. The size of the medication particles is another critical consideration, with formulations utilizing bases like olive oil or lanolin demonstrating enhanced absorption capabilities (Günther et al., 2020). This analysis (Table 1) showed that all the ointments were absorbed into the skin quickly. The oil content in formulated ointments is a deliberate formulation choice that contributes to their unique texture, facilitates the penetration of active ingredients, and provides occlusive properties to the skin. While the oily nature may feel greasy to some users, it is an essential aspect of ointments that enables the effective delivery of medications and promotes skin health.

#### 3.6.6. Topical sensitivity analysis

The ointments did not exhibit any adverse side effects during the evaluation period. No instances of skin inflammation, irritation, reddening, or allergic reactions were observed when the ointments were applied topically for one week.

#### 3.6.7. Microbial analysis

Microbiological analysis of the ointments revealed no detectable microbial contamination in either the oils or the ointments (Figure S2).

#### 3.6.8. Stability analysis over time

The ointments demonstrated robust stability, with no significant changes observed in physicochemical properties or microbial contamination under varying temperature conditions.

## 4. Conclusion

This research demonstrated the efficacy of EVOO-based cosmetic formulations enriched with GS and PP extracts. The ointments showed robust antioxidant activity through DPPH radical scavenging and contained key phenolic compounds (quercetin, catechin, and gallic acid). The enhanced water resistance properties made these ointments particularly suitable for skincare applications. The stability and microbiological analyses confirmed the robustness of the ointments, with no significant microbial contamination or degradation of key physicochemical properties over a six-month period. Notably, the use of beeswax and olive oil as base ingredients contributed to the stability and moisturizing properties of the ointments, which were shown to effectively maintain their integrity when exposed to moisture. This work validates the potential of agricultural byproducts in sustainable cosmetic development, offering natural alternatives to synthetic ingredients. The successful utilization of GS and PP extracts opens new avenues for ecofriendly skincare product development, warranting further investigation for expanded cosmetic and therapeutic applications.

## Supplementary materials

Figure S1 Phytochemical screening of the phenolics (A) and flavonoids (B).

Figure S2 Results of the microbial analysis for the oils and ointments.

## Figures and Tables

**Table 1:** R_f_ values of the DMSO-GS and PP-EVOO extracts, and standards, and refractive index and duration of absorption by the skin of the GS and PP-infused EVOO.

Oil samples	R_f_	Refractive index at 23 °C	Duration of absorption by skin (s) per 1 cm
**GGS**	0.94/0.9 /0.84 /0.26	1.4665	24.16 ± 0.66
**RGS**	0.94/0.86 /0.78 /0.28	1.4663	22.33 ± 1.11
**PP**	0.96/0.89 /0.82 /0.34	1.4664	15.66 ± 0.44
**EVOO**	0.94/0.87 /0.81 /0.33	1.4659	29.68 ± 1.39
**Quercetin**	0.86	/	/
**Catechin**	0.78	/	/
**Rutin**	0.24	/	/
**Gallic acid**	0.7	/	/

GGS: Green grape seeds, RGS: Red grape seeds, PP: Pomegranate peels, EVOO: Extra virgin olive oil.

**Table 2:** Fatty acid profiles of the EVOO and GS and PP-infused EVOO as a percentage of the total fatty acids quantified.

Compound Name	Retention time				Area%				Similarity			
	EVOO	GGS	RGS	PP	EVOO	GGS	RGS	PP	EVOO	GGS	RGS	PP
**Palmitoleic acid C16:1**	16.149	16.149	16.154	16.157	1.09	1.00	0.82	0.79	96	96	96	96
**Palmitic acid C16:0**	16.445	16.444	16.442	16.442	13.94	14.62	13.66	12.95	96	96	96	96
**Linoleic acid C 18:2**	18.811	18.811	18.812	18.811	8.7	8.58	7.52	6.48	93	93	93	94
**Oleic acid C18:1**	18.921	18.920	18.912	18.902	61.46	66.76	70.17	72.49	95	95	95	95
**Stearic acid C18:0**	19.205	19.204	19.209	19.208	3.43	3.51	3.05	2.67	95	95	96	96
2,6,10,15,19,23-Pentamethyl-2,6,18,22-tetracosatetraen-10,15-diol	29.147	/	/	/	1.10	/	/	/	96	/	/	/
