# Supplementary figures and images for: Enhanced formulation and comprehensive analysis of novel natural ointments with grape seed and pomegranate peel infused in olive oil
Source: Turk J Biol. 2024 Dec 16;49(1):28–39. doi: 10.55730/1300-0152.2721 (PMC11913359; doi:10.55730/1300-0152.2721)

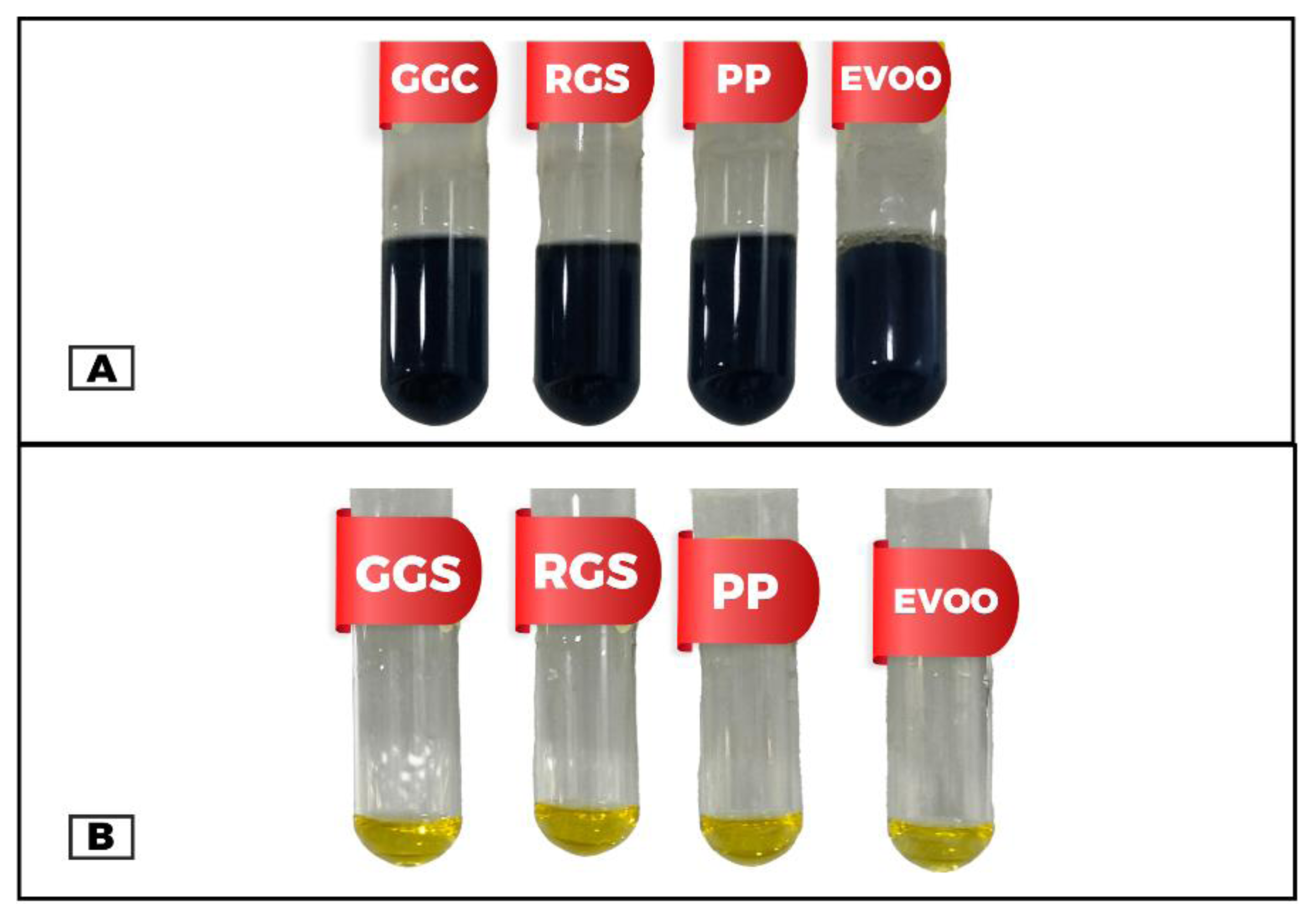

Supplement: Figure S1 — Phytochemical screening of the phenolics (A) and flavonoids (B). [file tjb-49-01-28s1.tif]

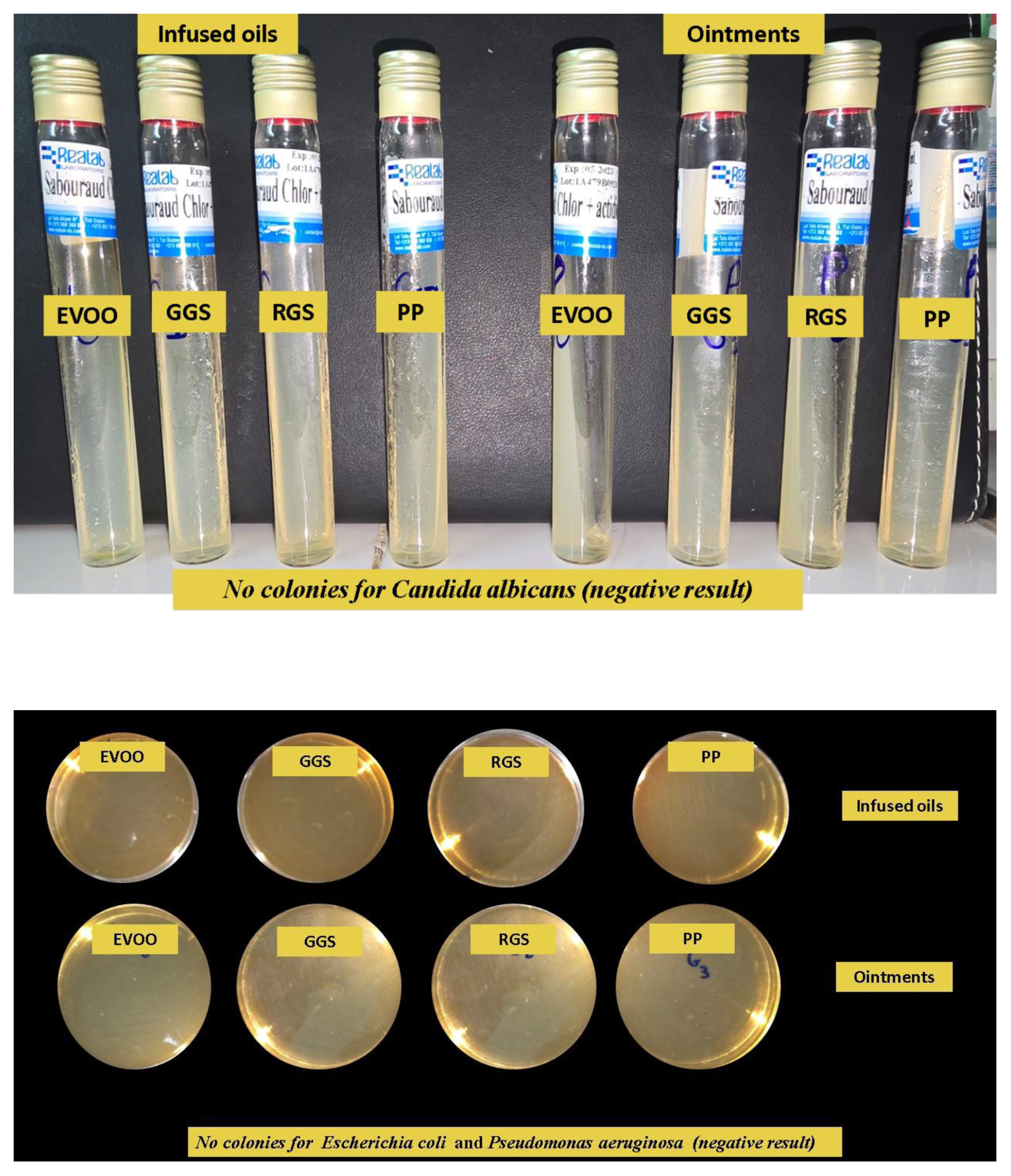

Supplement: Figure S2 — Results of the microbial analysis for the oils and ointments. [file tjb-49-01-28s2.tif]
